# Altered hormonal and autonomic nerve responses to hypo- and hyperglycaemia are found in overweight and insulin-resistant individuals and may contribute to the development of type 2 diabetes

**DOI:** 10.1007/s00125-020-05332-z

**Published:** 2020-11-26

**Authors:** Martin H. Lundqvist, Kristina Almby, Urban Wiklund, Niclas Abrahamsson, Prasad G. Kamble, Maria J. Pereira, Jan W. Eriksson

**Affiliations:** 1 Department of Medical Sciences, Uppsala University, Uppsala, Sweden; 2 Department of Radiation Sciences, Biomedical Engineering, Umeå University, Umeå, Sweden

**Keywords:** ACTH, Central nervous system, Cortisol, Diabetes, Glucoregulatory hormones, Glucose, Insulin resistance, Obesity

## Abstract

**Aims/hypothesis:**

Results from animal models and some clinical work suggest a role for the central nervous system (CNS) in glucose regulation and type 2 diabetes pathogenesis by modulation of glucoregulatory hormones and the autonomic nervous system (ANS). The aim of this study was to characterise the neuroendocrine response to various glucose concentrations in overweight and insulin-resistant individuals compared with lean individuals.

**Methods:**

Overweight/obese (HI, *n* = 15, BMI ≥27.0 kg/m^2^) and lean (LO, *n* = 15, BMI <27.0 kg/m^2^) individuals without diabetes underwent hyperinsulinaemic euglycaemic–hypoglycaemic clamps and hyperglycaemic clamps on two separate occasions with measurements of hormones, Edinburgh Hypoglycaemic Symptom Scale (ESS) score and heart rate variability (HRV). Statistical methods included groupwise comparisons with Mann–Whitney *U* tests, multilinear regressions and linear mixed models between neuroendocrine responses and continuous metabolic variables.

**Results:**

During hypoglycaemic clamps, there was an elevated cortisol response in HI vs LO (median ΔAUC 12,383 vs 4793 nmol/l × min; *p* = 0.050) and a significantly elevated adrenocorticotropic hormone (ACTH) response in HI vs LO (median ΔAUC 437.3 vs 162.0 nmol/l × min; *p* = 0.021). When adjusting for clamp glucose levels, obesity (*p* = 0.033) and insulin resistance (*p* = 0.009) were associated with elevated glucagon levels. By contrast, parasympathetic activity was less suppressed in overweight individuals at the last stage of hypoglycaemia compared with euglycaemia (high-frequency power of HRV, *p* = 0.024). *M* value was the strongest predictor for the ACTH and P_HF_ responses, independent of BMI and other variables. There was a BMI-independent association between the cortisol response and ESS score response (*p* = 0.024). During hyperglycaemic clamps, overweight individuals displayed less suppression of glucagon levels (median ΔAUC −63.4% vs −73.0%; *p* = 0.010) and more suppression of sympathetic relative to parasympathetic activity (low-frequency/high-frequency power, *p* = 0.011).

**Conclusions/interpretation:**

This study supports the hypothesis that altered responses of insulin-antagonistic hormones and the ANS to glucose fluctuations occur in overweight and insulin-resistant individuals, and that these responses are probably partly mediated by the CNS. Their potential role in development of type 2 diabetes needs to be addressed in future research.

**Figure:**
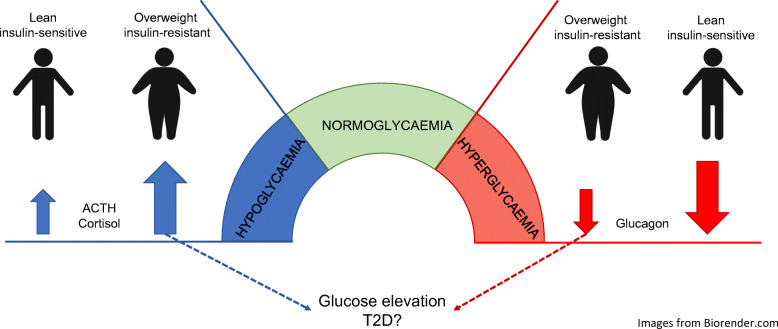
Graphical abstract

**Supplementary Information:**

The online version contains peer-reviewed but unedited supplementary material available at 10.1007/s00125-020-05332-z.

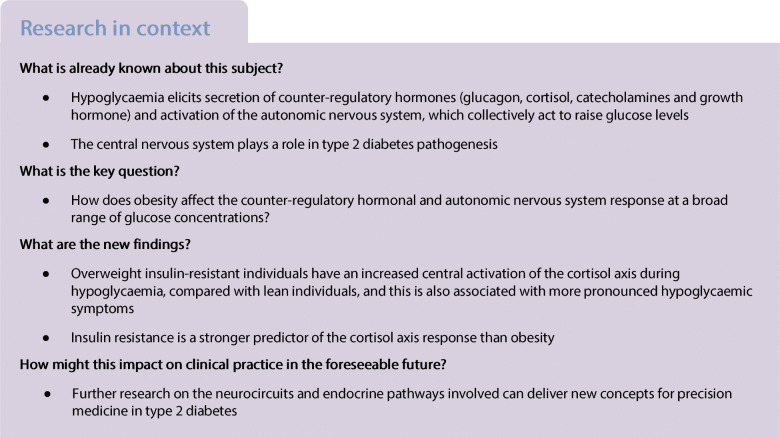



## Introduction

Since the 19th century, our understanding of glucose regulation and the pathogenesis of diabetes has mainly been based on processes in peripheral tissues, in particular the islets of Langerhans in the pancreas and target organs of insulin action such as the liver, muscle and adipose tissue. The ability of beta cells to detect and respond to varying glucose concentrations by modulating the secretion of insulin has long been considered as the major factor maintaining glucose homeostasis and explaining glucose dysregulation in diabetes. However, the importance of other glucose-regulating hormones, most notably glucagon, in diabetes development has been increasingly emphasised [1].

Hypoglycaemia elicits a typical response consisting of secretion of counter-regulatory hormones (glucagon, cortisol, catecholamines and growth hormone) and activation of the autonomic nervous system (ANS), which collectively act to raise glucose levels [2, 3]. Dysregulation in some of these hormonal and neural systems has been demonstrated in type 2 diabetes, prediabetes and obesity, suggesting a possible role in the development of type 2 diabetes [4–10].

Besides a relative insulin deficiency, hyperglucagonaemia is another hallmark of type 2 diabetes in humans. This has most clearly been demonstrated postprandially [4–6], whereas evidence of hyperglucagonaemia during fasting and experimental hypoglycaemia in obesity and type 2 diabetes is conflicting [7, 8, 11–14]. A biphasic glucagon response to different glucose levels has been described in mouse islets in vitro, with increased secretion in hyperglycaemic as well as hypoglycaemic conditions [15] but there is no evidence of this phenomena in human islets to our knowledge. The response of cortisol, adrenocorticotropic hormone (ACTH) and catecholamines to hypoglycaemia is reportedly, albeit inconsistently, augmented in obesity and/or type 2 diabetes, whereas basal levels have not been significantly different compared with healthy controls [7–10, 16, 17]. The growth hormone response to hypoglycaemia, on the other hand, has been consistently shown to be attenuated in obese individuals [18, 19]. Heart rate variability (HRV) studies have found evidence of lower parasympathetic activity in obesity, insulin resistance and type 2 diabetes, whereas sympathetic activity is not consistently altered [20–22]. The HRV response to hypoglycaemia in obesity and type 2 diabetes vs healthy individuals has not been thoroughly studied.

The brain is highly involved in the coordination of the counter-regulatory response to hypoglycaemia [2] and a ‘brain -centric’ model for glucose regulation has been proposed by some investigators [23]. According to this model, the brain senses glucose levels and mounts responses to deviations from a setpoint, much like a thermostat. Indeed, neurons that react to both high and low levels of glucose have been identified in the central nervous system (CNS). These neurons are most prevalent in the hypothalamus and the brain stem and project to other neurons involved in the regulation of hormonal axes and ANS activity [10].

We and others have performed hypoglycaemic clamps in obese individuals before and after gastric bypass surgery [24, 25]. After surgery, the responses of counter-regulatory hormones and sympathetic activity were markedly attenuated. Since asymptomatic hypoglycaemia is common after bariatric surgery, one possible explanation for this finding is that there is a post-surgery resetting of glucose regulation towards the hypoglycaemic range. Alternatively, it could reflect a normalisation of an exaggerated counter-regulatory response a priori in obese individuals. Such changes in the ‘glycaemic setpoint’ might involve altered glucose sensing and regulation by the CNS and may, in the long term, contribute to the development of type 2 diabetes, which is strongly associated with obesity.

In this study, to further elucidate regulation of insulin-antagonistic neurohormonal responses, we performed hyperinsulinaemic euglycaemic–hypoglycaemic clamps as well as hyperglycaemic clamps in individuals with varying BMI and degrees of insulin resistance. The objective was to investigate differences in the secretion of glucose-regulating hormones and ANS activity across a broad range of defined glucose concentrations. We also aimed to dissect the impact of obesity, insulin resistance and chronic dysglycaemia, respectively, on the perturbations of these neuroendocrine glucose-regulatory responses.

## Methods

### Participants

This study was conducted at the Uppsala University Hospital and the Department of Medical Sciences at Uppsala University. Participants were aged 18–60 years with a BMI of 18.5–50 kg/m^2^. They were recruited by advertisements in newspapers and public spaces, after previously participating in other studies or attending the outpatient obesity unit. Exclusion criteria were as follows: diagnosis of diabetes, endocrine or other diseases that could influence the results or the participants’ ability to participate in the study; use of medication with metabolic side effects; planned or ongoing pregnancy; and significant substance abuse. In the recruitment process, we aimed for a wide distribution of BMI. Participants were recruited across a wide range of BMI, aiming to have a representation of lean (18.5–24.9 kg/m^2^), overweight or mildly obese (25.0–34.9 kg/m^2^) and severely obese (35.0–50.0 kg/m^2^) individuals.

### Study design

Participants underwent a stepwise hyperinsulinaemic euglycaemic–hypoglycaemic clamp and a stepwise hyperglycaemic clamp on two occasions separated by 1–5 weeks. The order of the two clamps was randomised 1:1 in blocks of four subjects, and this was not blinded. At each visit, anthropometrics were obtained and body composition was assessed using bioimpedance (Tanita body composition analyzer, BC-418; Tanita Corporation, Tokyo, Japan). Heart rate and HRV were measured during both visits with a custom-made single-channel ECG recording system (developed at Biomedical engineering, R&D, University Hospital Umeå, Sweden). Baseline blood samples were drawn, after participants had fasted for at least 10 h overnight at both visits, just before the start of each clamp at approximately 09:00 hours.

**Fig. 1:**
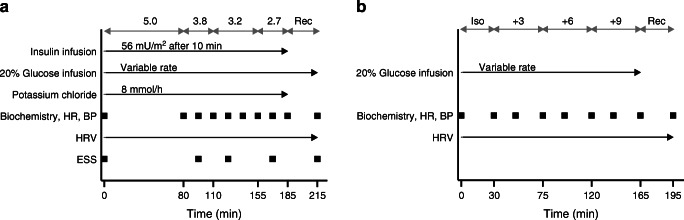
Clamp procedures during hypoglycaemic (**a**) and hyperglycaemic (**b**) clamps. Black squares indicate times at which measurements were taken. Target glucose levels (mmol/l) are indicated by the grey arrows at the top. ‘Biochemistry’ includes concentrations of glucose, glucagon, cortisol, ACTH, growth hormone, NEFA and glycerol; HR, heart rate; Iso, isoglycaemic phase of hyperglycaemic clamp; Rec, recovery

The hyperinsulinaemic euglycaemic–hypoglycaemic clamps (henceforth denoted hypoglycaemic clamps), depicted in Fig. 1a, were performed as previously described [26] and modified by our group through previous studies [24]. At 0 min, simultaneous infusions of insulin (56 mU m^−2^ min^−1^ after 10 min priming), potassium chloride (8 mmol/h) and glucose (200 mg/ml) were started. The glucose infusion rate (GIR) was adjusted to initially achieve a glucose level of 5.0 mmol/l followed by a stepwise lowering towards a nadir of 2.7 mmol/l. At 185 min, the insulin infusion was terminated and glucose was infused at a fixed rate of 200 mg kg^-1^ h^-1^ (recovery phase). The stepwise hyperglycaemic clamp protocol (Fig. 1b) was inspired by previously described two-step hyperglycaemic clamps [27, 28]. A variable glucose infusion (200 mg/ml) was administered to raise plasma glucose above the fasting level in three steps. At 165 min the glucose infusion was terminated to allow glucose levels to normalise. Glucose levels were analysed every 5 min during the clamps with blood drawn from an arterialised vein using a Contour Glucose Meter (Bayer Healthcare, Leverkusen, Germany).

During the clamps, hormonal and haemodynamic measurements were obtained at regular intervals. Single-channel ECG recordings were continuously made throughout each clamp. During hypoglycaemic clamps, participants were also asked to assess their hypoglycaemic symptoms according to the Edinburgh Hypoglycaemia Symptom Scale (ESS) [29, 30]. Further details are provided in electronic supplementary material (ESM) Methods.

### Biochemical measurements

Samples were analysed immediately or frozen at −80°C until analysis. Glucagon ELISA, NEFA fluorometric and glycerol colourimetric assays were analysed at the Clinical Diabetes Research Laboratory. All other analyses were performed at the Department of Clinical Chemistry at the Uppsala University Hospital, Sweden using a hexokinase method for glucose and immunoassays for hormones. Details of the analyses are provided in ESM Methods.

### HRV analysis

ECG recordings were automatically processed and manually inspected. The total spectral power (P_TOT_), low-frequency power (P_LF_, 0.04–0.15 Hz) and high-frequency power (P_HF_, 0.15–0.50 Hz) were calculated. P_HF_ mainly reflects the parasympathetic part of cardiac autonomic modulation, while P_LF_ reflects a combination of sympathetic and parasympathetic activity. The ratio P_LF_/P_HF_ is used as a proxy for balance between sympathetic and parasympathetic activity [31]. The HRV analysis was performed using Matlab Software version R2019a (MathWorks, Natick, MA, USA). More details are provided in ESM Methods.

### Statistical analysis

This was an exploratory study, and no formal power calculation for sample size selection was performed. The primary endpoint was the glucagon levels during experiments, including hypo- and hyperglycaemia. As estimated from previous studies, there is more than 80% power to detect 25% differences in glucagon levels between groups with 15 participants each, assuming an inter-subject CV of 0.20 and a two-sided α of 0.05; the same estimate also applies for cortisol levels [24, 25, 32–35]. In the analyses, participants were allocated to two equally large groups, lean (LO) and overweight/obese (HI) with cut-off at the median BMI. Mann–Whitney *U* tests were consistently used for groupwise comparisons of continuous variables and Fisher’s exact test for categorical variables. *p* values <0.05 were considered significant.

Spearman’s Rank correlation analyses and multilinear regression analyses were performed on pooled data for all participants between neuroendocrine response variables (hormones and HRV indices) during the hypoglycaemic and hyperglycaemic phases and candidate predictors.

To describe hormonal responses vs achieved glucose levels, scatterplots of hormone levels at the end of each clamp stage vs the mean glucose for the preceding 20 min were used for visual presentation, and linear mixed models were used for statistical inference. All statistical methods are described in detail in ESM Methods.

All analyses were performed in SPSS for Mac version 25 (IBM Corp., Armonk, NY, USA). Figures and graphs were made using GraphPad Prism version 8.3.0 (GraphPad Software, San Diego, CA, USA).

### Ethics

All study procedures were performed in accordance with the Declaration of Helsinki. The local Research Ethics Committee of Uppsala gave their approval (DNR 2017/550). The participants received oral and written information about the study and signed an informed consent form.

## Results

Thirty participants were included, with a median BMI of 27.0 kg/m^2^, defining the cut-off between groups LO and HI. Participant characteristics from visit 1 (baseline) are shown in Table 1. The distribution of sex and age was similar in the groups. Fasting glucose levels were similar in the groups while HI had higher HOMA-IR than LO. One participant in each group had fasting glucose above 7.0 mmol/l but HbA_1c_ was normal. One participant in HI did not complete the hyperglycaemic clamp because of problems with maintaining venous access and was omitted from the analysis. For four participants in HI, insulin infusion rates had to be increased during hypoglycaemic clamps because of difficulties in obtaining target glucose levels. HRV analysis was not possible due to imperfect ECG recordings for four participants undergoing hypoglycaemic clamps and two undergoing hyperglycaemic clamps (all group LO), and these participants were omitted from these analyses.

**Table 1:** Characteristics of participants

Variable	LO (BMI <27.0 kg/m^2^; *n*=15)	HI (BMI ≥27.0 kg/m^2^; *n*=15)	*p* value (HI vs LO)
Age, years	41 (30, 51)	43 (31, 54)	0.775
Sex, *n* male/*n* female	5/10	3/12	0.682
Weight, kg	70.0 (59.0, 82.0)	97.2 (86.1, 127.6)	<0.001***
BMI, kg/m^2^	23.4 (22.5, 26.0)	32.0 (28.9, 45.6)	<0.001***
Body fat, %	22.1 (20.7, 29.4)	42.0 (38.2, 51.2)	<0.001***
Waist/hip ratio	0.83 (0.77, 0.90)	0.94 (0.90, 0.99)	<0.001***
ESS score	12 (12, 17)	13 (12, 16)	0.713
Resting heart rate, bpm	60 (56, 68)	65 (56, 76)	0.412
Systolic BP, mmHg	115 (110, 130)	128 (120, 135)	0.041*
Diastolic BP, mmHg	78 (74, 85)	84 (78, 90)	0.033*
HbA_1c_, mmol/mol	34 (31, 34)	35 (32, 37)	0.233
HbA_1c_, %	5.3 (5.0, 5.3)	5.4 (5.1, 5.5)	0.233
Fasting plasma glucose, mmol/l	5.5 (5.3, 5.9)	5.8 (5.4, 6.2)	0.187
Serum C-peptide, nmol/l	0.6 (0.5, 0.8)	1.1 (0.6, 1.3)	0.001**
Serum insulin, pmol/l	31.9 (24.3, 55.6)	79.9 (41.0, 152.8)	0.001**
HOMA-IR	1.14 (0.86, 1.96)	3.27 (1.42, 5.21)	0.001**
Plasma cholesterol, mmol/l^a^	4.6 (3.9, 5.0)	4.4 (3.7, 5.2)	0.872
Plasma LDL, mmol/l	2.5 (2.2, 2.7)	2.9 (2.4, 3.7)	0.067
Plasma HDL, mmol/l	1.50 (1.20, 1.90)	0.91 (0.86, 1.10)	<0.001***
Plasma triacylglycerols, mmol/l	0.65 (0.51, 0.76)	0.99 (0.72, 1.29)	0.009**
Plasma glucagon, pmol/l	8.9 (6.5, 12.3)	10.5 (9.0, 13.1)	0.486
Serum cortisol, nmol/l	257 (197, 338)	170 (127, 269)	0.037*
Plasma ACTH, pmol/l	2.3 (2.1, 4.4)	2.5 (1.8, 4.6)	0.775
Plasma GH, μg/l	1.08 (0.15, 4.20)	0.37 (0.07, 1.06)	0.126
Plasma NEFA, μmol/l	182.0 (132.7, 232.1)	258.6 (212.8, 553.5)	0.002**
Plasma glycerol, μmol/l	63.7 (53.2, 82.3)	78.7 (65.8, 102.8)	0.050

Data are presented as median (25th percentile, 75th percentile) and were obtained at the first visit (or second if missing at first)

^a^Samples are missing for eight participants

*p<0.05, **p<0.01 and ***p<0.001

GH, growth hormone

Complete clamp measurements are provided in ESM Tables 1 and 2.

### Hypoglycaemic clamps

#### Metabolism

**Fig. 2:**
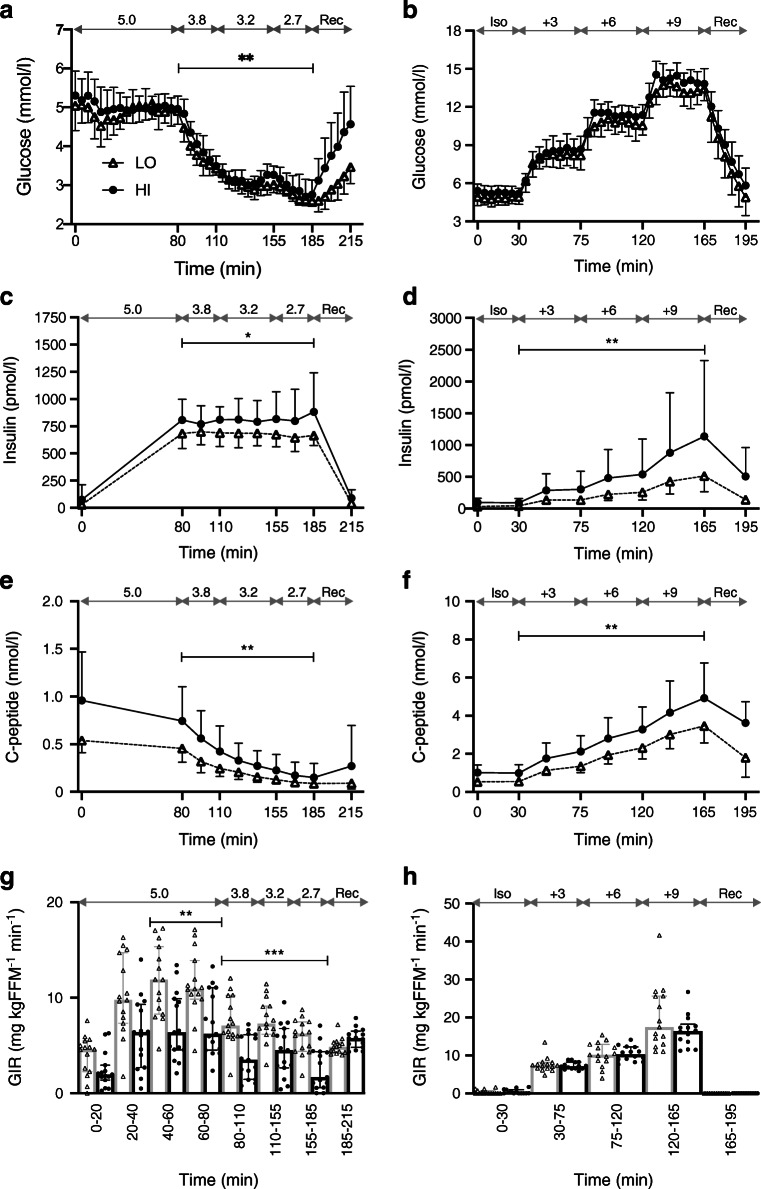
Levels of glucose measured by bedside glucometer (**a**, **b**), insulin (**c**, **d**), C-peptide (**e**, **f**) and GIR/FFM (**g**, **h**). Data are presented as geometric means ± geometric SD (**a**–**f**) and medians ± IQRs, as well as individual values (**g**, **h**). Data from hypoglycaemic clamps (**a**, **c**, **e**, **g**) and hyperglycaemic clamps (**b**, **d**, **f**, **h**) are shown. Target glucose levels (mmol/l) are indicated by the grey double-headed arrows. Black circles and solid lines, HI (BMI ≥27.0 kg/m^2^; *n*=15 in **a**, **c**, **e**, **g** and *n*=14 in **b**, **d**, **f**, **h**); white triangles and dashed lines, LO (BMI <27.0 kg/m^2^; *n*=15 in all panels); in (**g**, **h**), black circles and bars, HI; white triangles and grey bars, LO. Significance estimates refer to comparisons of the AUC (**a**–**f**) or total amount of infused glucose (**g**) for the indicated time periods between groups HI and LO: *p<0.05, **p<0.01, ***p<0.001. Groupwise comparison of central laboratory glucose measures gave similar results as for glucometer measures, which are reported here owing to more frequent sampling. Complete measurements are provided in ESM Table 1. Iso, isoglycaemic phase of hyperglycaemic clamp; Rec, recovery

Glucose levels were higher in HI vs LO during hypoglycaemia, and rose faster during the recovery phase (Fig. 2a and ESM results). The GIR per kg fat-free mass (FFM) was consistently lower in HI vs LO throughout the clamp, and the *M* value (GIR/FFM from 40 min to 80 min) was significantly lower (*p* = 0.007, Fig. 2g and ESM Table 1). Fasting NEFA (*p* = 0.002) and glycerol (*p* = 0.050) levels were higher in HI vs LO. In both groups, they dropped similarly during hyperinsulinaemia–euglycaemia and then stabilised during hypoglycaemia, with NEFA levels remaining higher (*p* = 0.004) in HI vs LO (Table 1, ESM Table 1).

#### Hormonal response

**Fig. 3:**
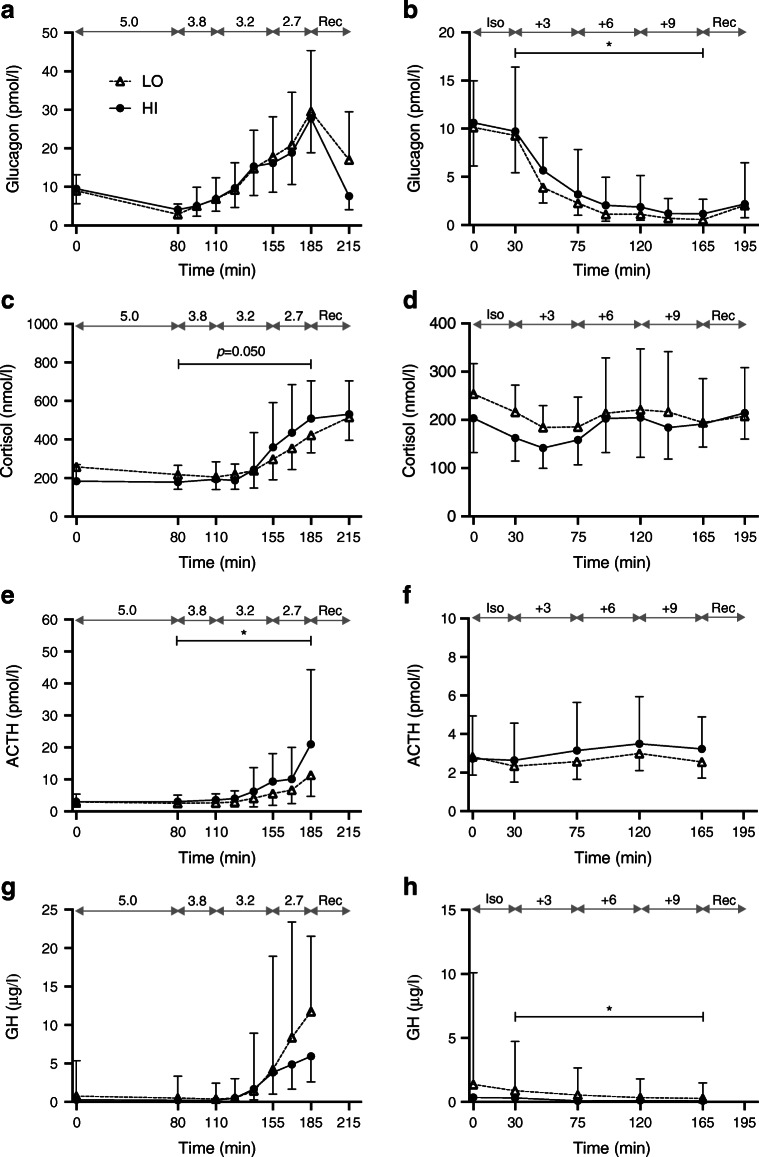
Levels of counter-regulatory hormones. Data are presented as geometric means ± geometric SD. Data from hypoglycaemic clamps (**a**, **c**, **e**, **g**) and hyperglycaemic clamps (**b**, **d**, **f**, **h**) are shown. Target glucose levels (mmol/l) are indicated by the grey double-headed arrows. Black circles and solid lines, HI (BMI ≥27.0 kg/m^2^; *n*=15 in **a**, **c**, **e**, **g** and *n*=14 in **b**, **d**, **f**, **h**); white triangles and dashed lines, LO (BMI <27.0 kg/m^2^; *n*=15 in all panels). Significance estimates refer to comparison of ΔAUC (**b**, **c**, **e**) or AUC (**h**) between groups HI and LO: *p<0.05. Complete measurements are provided in ESM Table 1. GH, growth hormone; Iso, isoglycaemic phase of hyperglycaemic clamp; Rec, recovery

Insulin and C-peptide levels were higher in HI vs LO (Fig. 2c,e). Glucagon levels did not differ between the groups (Fig. 3a). Cortisol levels were lower at baseline and the change in AUC (ΔAUC) was higher in HI vs LO (median 12,383 vs 4793 nmol/l × min; *p* = 0.050), although not significantly (Table 1, Fig. 3c). ACTH did not differ at baseline and rose to higher levels in HI vs LO (median ΔAUC 437.3 vs 162.0 nmol/l × min; *p* = 0.021) (Fig. 3e). Growth hormone levels were lower in HI vs LO at baseline (*p* = 0.126) as well as during hypoglycaemia (*p* = 0.250) but the differences were not significant (Table 1, Fig. 3g).

#### Haemodynamic measurements, HRV and symptoms

Baseline heart rate did not differ between the groups; systolic and diastolic BP were higher in HI but they behaved similarly during the clamps (Table 1, ESM Table 2).

**Fig. 4:**
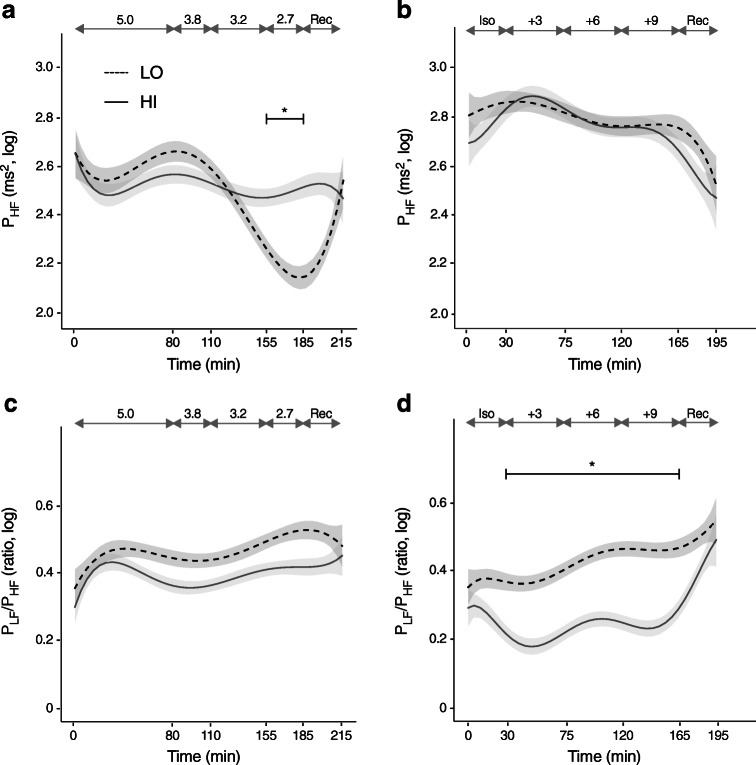
HRV spectral components. Data have been log-transformed (base 10) and are presented as means and SEM (shaded area). Data from hypoglycaemic clamps (**a**, **c**) and hyperglycaemic clamps (**b**, **d**) are shown. Target glucose levels (mmol/l) are indicated by the grey double-headed arrows. Solid lines, HI (BMI ≥27.0 kg/m^2^; *n*=15 in **a**, **c** and *n*=14 **b**, **d**); dashed lines, LO (BMI <27.0 kg/m^2^; *n*=11 in **a**, **c** and *n*=13 in **b**, **d**). Significance estimates refer to comparison of mean values of the indicated time period between groups HI and LO adjusted for the mean values during the euglycaemic phase (**a**) or the isoglycaemic phase (**d**): *p<0.05. Complete measurements are provided in ESM Table 2. Iso, isoglycaemic phase of hyperglycaemic clamp; Rec, recovery

The HRV indices did not differ significantly between the groups during the euglycaemic phase. During hypoglycaemia, compared with euglycaemia, the RR interval, P_TOT_, P_LF_ and P_HF_ fell to a lesser degree in HI vs LO and in the last stage of hypoglycaemia there was a significant group difference in all these responses (*p* = 0.024 for P_HF_) (Fig. 4a and ESM Table 2). The P_LF_/P_HF_ ratio did not, however, differ between the groups (Fig. 4c, ESM Table 2). Participants in HI reported more pronounced symptoms than those in LO but the difference was not significant (*p* = 0.126 for both peak and ΔESS scores; Table 2).

**Table 2:** ESS scores during hypoglycaemic clamps

Measurement	LO	HI	*p* value (HI vs LO)
ESS score			
Peak	18 (15, 25)	21 (20, 23)	0.126
ΔPeak–baseline	5 (2, 9)	7 (5, 9)	0.126
Auto			
Peak	7 (6, 14)	11 (9, 12)	0.217
ΔPeak−baseline	2 (2, 6)	5 (2, 7)	0.233
Neuro			
Peak	8 (6, 9)	9 (7, 10)	0.325
ΔPeak−baseline	2 (2, 3)	3 (2, 4)	0.624
Nausea			
Peak	2 (2, 3)	3 (2, 4)	0.412
ΔPeak−baseline	0 (0, 1)	1 (0, 1)	0.567

Data are presented as median (25th percentile, 75th percentile)

Auto, autonomic domain of ESS; Neuro, neuroglycopenic domain of ESS

#### Associations between neuroendocrine responses and metabolic phenotype

**Fig. 5:**
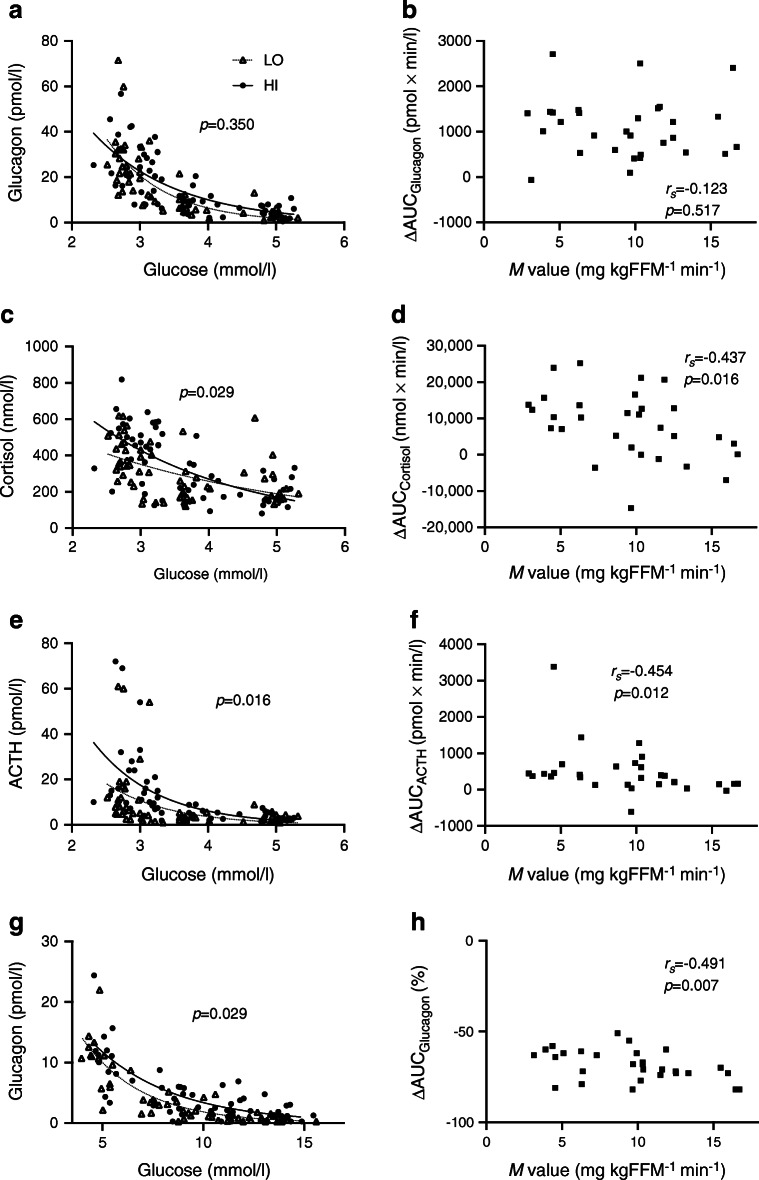
Scatterplots of hormone levels vs glucose levels (**a**, **c**, **e**, **g**) and of ΔAUC of hormones vs *M* value (**b**, **d**, **f**, **h**). Hormone levels were measured at the end of each clamp stage vs mean glucometer glucose levels for the preceding 20 min during hypoglycaemic clamps (**a**, **c**, **e**) or hyperglycaemic clamps (**g**). *p* values refer to estimates of group differences (**a**, **e**, **g**) or interaction group × glucose (**c**) in linear mixed models. Black circles, HI (BMI ≥27.0 kg/m^2^; *n*=15 in **a**, **c**, **e** and *n*=14 in **g**); white triangles, LO (BMI <27.0 kg/m^2^; *n*=15 in **a**, **c**, **e**, **g**); solid lines, exponential regression curve for HI; dotted lines, exponential regression curve for LO; black squares, ΔAUC of the hypoglycaemic phase (80–185 min) (**b**, **d**, **f**; *n*=30) or the hyperglycaemic phase (30–165 min) (**h**; *n*=29) for all participants vs the *M* value; *r*_*s*_*,* Spearman’s rho coefficient with corresponding *p* value

Main results are displayed in Tables 3, 4. Scatterplots of ΔAUC for glucagon, cortisol and ACTH vs *M* value are displayed in Fig. 5 (b,d,f).

**Table 3:** Correlations between neuroendocrine responses and metabolic phenotype

Variable^a^	Hypoglycaemic clamps						Hyperglycaemic clamps					
	Glucagon	Cortisol	ACTH	GH	P_HF_	P_LF_/P_HF_	Glucagon	Cortisol	ACTH	GH	P_HF_	P_LF_/P_HF_
Age	−0.156 (0.410)	−0.256 (0.171)	0.191 (0.312)	−0.07 (0.713)	0.383 (0.053)	−0.224 (0.270)	0.118 (0.542)	−0.116 (0.549)	0.051 (0.793)	−0.002 (0.992)	0.319 (0.105)	−0.331 (0.092)
Sex	0.174 (0.357)	0.105 (0.583)	0.035 (0.855)	0.462 (0.01)*	0.268 (0.186)	−0.377 (0.057)	−0.074 (0.704)	−0.203 (0.291)	−0.406 (0.029)*	0.051 (0.794)	0.010 (0.959)	0.187 (0.349)
BMI	0.034 (0.858)	0.420 (0.021)*	0.507 (0.004)**	−0.171 (0.366)	0.570 (0.002)**	−0.337 (0.092)	0.542 (0.002)**	0.182 (0.344)	0.182 (0.345)	0.178 (0.355)	0.287 (0.147)	−0.530 (0.004)**
WHR	0.193 (0.307)	0.385 (0.036)*	0.520 (0.003)**	0.225 (0.231)	0.459 (0.018)*	−0.265 (0.191)	0.166 (0.391)	−0.038 (0.845)	−0.080 (0.679)	0.048 (0.804)	0.261 (0.189)	−0.397 (0.040)*
% Body fat	−0.010 (0.960)	0.241 (0.199)	0.393 (0.032)*	−0.358 (0.052)	0.301 (0.136)	−0.165 (0.421)	0.452 (0.014)*	0.259 (0.175)	0.304 (0.108)	0.152 (0.430)	0.213 (0.285)	−0.435 (0.023)*
Fasting glucose	−0.313 (0.092)	0.110 (0.562)	0.177 (0.349)	0.029 (0.991)	0.491 (0.011)*	−0.550 (0.004)**	0.404 (0.030)*	−0.318 (0.092)	−0.273 (0.152)	0.010 (0.961)	0.013 (0.948)	−0.076 (0.707)
HbA_1c_	−0.303 (0.104)	−0.049 (0.798)	0.127 (0.503)	−0.150 (0.430)	0.373 (0.061)	−0.261 (0.199)	0.329 (0.081)	−0.147 (0.446)	−0.158 (0.413)	−0.268 (0.160)	0.119 (0.554)	−0.243 (0.222)
*M* value	−0.123 (0.517)	−0.437 (0.016)*	−0.454 (0.012)*	−0.090 (0.638)	−0.716 (<0.001)***	0.527 (0.006)**	−0.491 (0.007)**	0.014 (0.941)	0.104 (0.592)	−0.191 (0.321)	−0.227 (0.255)	0.408 (0.034)*
HOMA-IR	0.146 (0.442)	0.503 (0.005)**	0.455 (0.012)*	−0.205 (0.276)	0.594 (0.001)**	−0.641 (<0.001)***	0.550 (0.002)**	−0.075 (0.698)	−0.284 (0.135)	0.104 (0.593)	−0.045 (0.825)	−0.243 (0.222)

Data are presented as Spearman’s Rho coefficient, *r*_*s*_, with *p* values in parentheses

Data are pooled from all participants (*n*=30 in hypoglycaemic clamps and *n*=29 in hyperglycaemic clamps)

Neuroendocrine responses are ΔAUC for hormones and Δmean for P_HF_ and P_LF_/P_HF_. This is described in more detail in ESM Methods

^a^Variables are defined in ESM Methods

*p<0.05, **p<0.01 and ***p<0.001

GH, growth hormone

**Table 4:** Multilinear regressions of neuroendocrine responses vs metabolic phenotype

Variable^a^	Hypoglycaemic clamps				Hyperglycaemic clamps	
	Cortisol	ACTH	P_HF_	P_LF_/P_HF_	Glucagon^b^	P_LF_/P_HF_
*R*^2^ (*p* value)	0.188 (0.061)	0.208 (0.043)*	0.484 (0.002)**	0.172 (0.236)	0.475 (0.003)**	0.146 (0.151)
BMI, β (*p* value)	−0.022 (0.931)	−0.396 (0.121)	0.143 (0.540)	−0.051 (0.864)	0.480 (0.029)*	−0.379 (0.153)
Fasting glucose, β (*p* value)	NA	NA	0.103 (0.570)	−0.261 (0.259)	−0.009 (0.957)	NA
*M* value, β (*p* value)	−0.449 (0.085)	−0.651 (0.014)*	−0.527 (0.031)*	0.178 (0.546)	−0.482 (0.061)	0.004 (0.988)

Data are pooled from all participants (*n*=30 in hypoglycaemic clamps and *n*=29 in hyperglycaemic clamps)

Neuroendocrine responses are ΔAUC for hormones and Δmean for P_HF_ and P_LF_/P_HF_. This is described in more detail in ESM Methods

^a^Variables are defined in ESM Methods

^b^Adjusted for AUC_Insulin_

*p<0.05 and **p<0.01

β, standardised coefficient; NA, not applicable/not analysed

Both obesity indices and insulin resistance indices were associated with a higher response of the cortisol axis and an HRV response indicative of less parasympathetic suppression. Insulin resistance was also associated with less sympathetic activation. The fasting glucose was correlated only with the HRV responses, in the same direction as obesity and insulin resistance. The growth hormone response only correlated with sex (higher in women). ΔESS scores were positively correlated with the cortisol and ACTH response (ESM Table 3, ESM Fig. 1).

In multilinear regressions, *M* value was the strongest predictor for the ACTH and P_HF_ responses, independent of BMI and other variables (Table 4). Excluding one outlier, the cortisol response had a positive association with ΔESS, independent of BMI (ESM Table 3, ESM Fig. 1).

#### Hormonal response vs achieved glucose levels

Figure 5 displays scatterplots of glucagon (Fig. 5a), cortisol (Fig. 5c) and ACTH (Fig. 5e) vs achieved mean glucose levels of each participants. The levels of all three hormones appear higher at the same glucose levels in HI vs LO. Although the group difference for glucagon was not significant during hypoglycaemia, BMI (*p* = 0.033) was associated with higher glucagon levels and *M* value (*p* = 0.009) with lower glucagon levels in linear mixed models.

### Hyperglycaemic clamps

#### Metabolism

Glucose levels during hyperglycaemia were higher (but not significantly) in HI vs LO (*p* = 0.063) (Fig. 2b). The GIR per kg FFM did not differ between the groups (Fig. 2h). Glycerol and NEFA levels were generally higher in HI vs LO but the decreases during hyperglycaemia were similar between groups (ESM Table 1).

#### Hormonal response

Insulin and C-peptide were generally elevated in HI vs LO but the fold rise during hyperglycaemia was similar (Fig. 2d,f). The suppression of glucagon levels was attenuated during hyperglycaemia (median ΔAUC −63.4% vs −73.0%; *p* = 0.010) and overall levels were non-significantly higher (*p* = 0.085) in HI vs LO (Fig. 3b). Cortisol and ACTH levels did not differ between the groups; growth hormone levels were generally lower in HI vs LO but fell similarly during hyperglycaemia (Fig. 3d,f,h).

#### Haemodynamic measures and HRV

Heart rate did not differ between the groups. Systolic and diastolic BP were overall higher during hyperglycaemia in HI vs LO, although the differences did not reach significance (*p* = 0.089 and *p* = 0.111, respectively), and the trajectories during the clamp were similar (ESM Table 2).

None of the HRV indices differed significantly during the isoglycaemic phase. The P_LF_/P_HF_ ratio decreased during hyperglycaemia in HI but not in LO (*p* = 0.011) (Fig. 4b,d). Other HRV indices did not differ significantly between the groups (ESM Table 2).

#### Associations between neuroendocrine responses and metabolic phenotype

Main results are displayed in Tables 3 and 4. A scatterplot of ΔAUC for glucagon vs *M* value is displayed in Fig. 5h.

Obesity and insulin resistance measurements were positively correlated with less suppression of glucagon and more suppression of the P_LF_/P_HF_ response. In addition, fasting glucose was positively correlated with less glucagon suppression.

In multilinear regressions, BMI had the strongest association with the glucagon and P_LF_/P_HF_ response, although the associations were not statistically significant. When adjusting for insulin levels, the association between BMI and the glucagon response was significant however.

#### Hormonal response vs achieved glucose levels

The overall glucagon levels were higher in HI vs LO at any given glucose level and the hyperglycaemia-induced suppression was less marked (Fig. 5g).

## Discussion

In this study, we hypothesised that overweight and insulin-resistant individuals have a higher setpoint for homeostatic regulation of circulating glucose levels than lean individuals. This would be reflected by faster or elevated counter-regulatory (i.e. insulin-antagonistic) responses to hypoglycaemia and, vice versa, by delayed or attenuated suppression of such responses during hyperglycaemia. Such findings could suggest a role for neuroendocrine dysregulation in the pathogenesis of type 2 diabetes.

### Hypoglycaemia

The main finding from the hypoglycaemic clamps was an augmented responsiveness of the cortisol axis to hypoglycaemia among overweight and insulin-resistant participants compared with lean and more insulin-sensitive participants. This appears to be of central origin, involving hypothalamic and pituitary functions, since ACTH and cortisol responses were similarly elevated in the HI vs LO group.

While the elevated hypoglycaemic symptom scores in HI vs LO did not reach significance, there were significant, BMI-independent, associations between symptoms and the cortisol axis response, suggesting a causal connection between perceived glucopenia and the augmented cortisol axis response in overweight individuals. Undoubtedly, the anatomical bridge for this connection would be within the CNS. Therefore, an increased CNS sensing of hypoglycaemia in obese individuals is possible but obviously not proven. This would be expected to raise the glycaemic ‘setpoint’ for cortisol axis responses but the magnitude of this shift could not be exactly defined due to the limited sample size and experimental design. However, as visualised in Fig. 5(c,e), a physiological cut-off for hypoglycaemia of 3 mmol/l appears to be shifted to about 3.3 mmol/l in the HI vs LO group with respect to ACTH and cortisol responses. Moreover, the fact that the enhanced cortisol axis response was inversely associated with the *M* value, independent of BMI, points to a potential role in the development of insulin resistance. This may be further amplified by an increased local generation of cortisol in adipose tissue, and elevated tissue cortisol can be hypothesised to play a role in type 2 diabetes development [36]. Naturally, longitudinal studies of larger cohorts are needed to further support this hypothesis.

We evaluated sympathetic and parasympathetic nerve activity by HRV assessments. Given the increased symptoms of hypoglycaemia, an augmented sympathetic response in the overweight group might have been surmised. However, both obesity and, more clearly, insulin resistance was associated with a less dynamic ANS response to hypoglycaemia, characterised by less parasympathetic inhibition. Insulin resistance but not obesity was also associated with less sympathetic activation. Of interest, our group has previously reported that gastric bypass surgery was followed by an attenuated ANS response to hypoglycaemia [24]. We also reported that visceral adiposity [33] as well as insulin resistance [37] was associated with an increased sympathetic/parasympathetic ratio under normoglycaemic conditions. It should be acknowledged that HRV has limitations as a marker of ANS activity and has been questioned [38]. Although P_HF_ supposedly reflects parasympathetic activity, the P_LF_ represents both sympathetic and parasympathetic activity, and the P_LF_/P_HF_ ratio is utilised to reflect their relative contributions. Moreover, the importance and exact peripheral mechanisms of the two branches of the ANS with regards to glucose regulation is still uncertain [39–41]. The role of parasympathetic activity is particularly controversial. Apart from a proposed stimulation of glucagon secretion [40], our research group reported an unexpected rapid increase in insulin sensitivity following infusion of atropine [42], suggesting a paradoxical short-term effect by cholinergic pathways of the parasympathetic system to reduce peripheral glucose uptake. Thus, the attenuated inhibition of parasympathetic activity observed in overweight and insulin-resistant individuals during hypoglycaemia may potentially enhance the defence against hypoglycaemia and the maintenance of elevated everyday glucose levels. Catecholamine levels would be of interest in this context but they were presently not possible to analyse and were not considered as critical, since HRV assessments did not suggest any substantial group difference in sympathetic activity.

We found no differences in glucagon levels between groups during hypoglycaemia, nor were they associated with measures of obesity or insulin resistance. However, there were group differences in achieved glucose and insulin levels that may have underestimated differences in hormone responses. When adjusting for glucose levels in linear mixed models, both obesity and insulin resistance were indeed associated with significantly higher glucagon levels during hypoglycaemia. This is in concordance with a previous study showing augmented glucagon, ACTH and noradrenaline (norepinephrine) responses in obese individuals exposed to hypoglycaemia [7]. The more pronounced differences in their study may be due to use of the less specific RIA technique for glucagon measurement [32], greater BMI difference between groups and a younger and all-male study population.

The attenuation of the growth hormone response during hypoglycaemia in overweight individuals did not reach significance in our study, but has been observed in previous studies [18, 19].

### Hyperglycaemia

During the hyperglycaemic clamps, overweight and insulin-resistant participants displayed less suppression of glucagon than lean participants. This is in accordance with some previous studies [43–45] and may contribute to the development and progression of insulin resistance and potentially type 2 diabetes.

The cortisol responses to hyperglycaemia were highly variable between individuals and there were no consistent differences between groups. The initial decline in both groups is most likely explained by diurnal variations and the subsequent rise could represent a glucose-mediated stress response. A rise in cortisol levels after meals or an oral glucose load is well-established in previous work [46, 47].

We found markedly lower growth hormone levels during hyperglycaemia in overweight participants but glucose-mediated inhibition was similar to that in lean participants, which is somewhat different to findings of a previous study [48].

The P_LF_/P_HF_ ratio rose during hyperglycaemia but was significantly lower in the overweight group indicative of a decreased sympathetic relative to parasympathetic activity. This resembles findings during hypoglycaemic conditions. Thus, the sympathetic response to acute hyperglycaemia and hyperinsulinaemia appears to be impaired in overweight insulin-resistant individuals, perhaps because of adaptation to slightly elevated glucose levels.

### Limitations

There are some limitations to this study. First, as previously discussed, the minutely higher glucose and insulin levels achieved during clamps in overweight compared with lean participants may have affected insulin-antagonistic responses. Notably, this would mainly underestimate the differences in hormone levels found and, for completeness, we also adjusted for actual glucose levels in regression analyses. Second, while the elevated hormonal responses associated with obesity and insulin resistance are compatible with a hypothesised upward shift in glycaemic setpoint, our current data do not allow more than a very rough quantification of this shift. We plan analyses of data from pooled cohorts to address this. Third, the design may be underpowered to detect hypothetical effect sizes of interest. Fourth, several of the neuroendocrine alterations reported in this study were small in magnitude and the clinical implications need to be confirmed. Fifth, participants were recruited based on BMI rather than insulin resistance and the associations with neuroendocrine responses should be interpreted in the light of this. However, recruiting participants based on measures of insulin resistance or dysglycaemia (e.g. following OGTTs) would have markedly hampered feasibility. Finally, no adjustment for multiple comparisons was made. This was due to the exploratory nature of this work and also to biological interdependencies of the measured neurohormonal responses.

Overall, these findings are hypothesis-generating and need to be confirmed in larger studies.

### Conclusion

This study demonstrates that overweight insulin-resistant individuals compared with lean individuals have increased central activation of the cortisol axis during hypoglycaemia, associated with more pronounced hypoglycaemic symptoms. This suggests an increased CNS-mediated response to hypoglycaemia. The finding that insulin resistance, more than obesity, is associated with the cortisol axis response is compatible with a causal role of the neurohormonal responses for the development of dysglycaemia and potentially type 2 diabetes. Furthermore, an attenuated suppression during hyperglycaemia and, to a lesser extent, an augmented glucagon rise during hypoglycaemia seem to be features of both insulin resistance and obesity. The anatomical sites, such as brain, pancreas or both, involved in this dysregulation remain to be elucidated in onward studies. By contrast, there is an attenuation of autonomic nerve responses to glucose fluctuations in overweight and insulin-resistant individuals that may reflect a less dynamic sympathetic and parasympathetic regulation, which in the long term may potentially contribute to dysglycaemia.

Taken together, altered insulin-antagonistic responses, including the cortisol axis, glucagon and ANS, in obese insulin-resistant individuals may contribute to the development of long-term dysglycaemia and, hypothetically, also type 2 diabetes. These perturbations may involve glucoregulatory functions of the brain shifting the ‘glycaemic setpoint’ for glucose-regulating hormones upwards. Our ongoing and future work will include individuals with type 2 diabetes and also the use of neuroimaging techniques to assess regional brain responses to hypo- and hyperglycaemia during diabetes development.

## Supplementary Information

ESM (PDF 643 kb)

## Data Availability

Further information about data and resources will be provided upon request to the corresponding author.

## Abbreviations

ACTH: Adrenocorticotropic hormone
ANS: Autonomic nervous system
CNS: Central nervous system
ESS: Edinburgh Hypoglycaemia Symptom Scale
FFM: Fat-free mass
GIR: Glucose infusion rate
HRV: Heart rate variability
LO: Lean
HI: Non-diabetic overweight/obese
P_HF_: High-frequency power
P_LF_: Low-frequency power
P_TOT_: Total spectral power
