# Supplementary material for: Altered hormonal and autonomic nerve responses to hypo- and hyperglycaemia are found in overweight and insulin-resistant individuals and may contribute to the development of type 2 diabetes
Source: Diabetologia. 2020 Nov 26;64(3):641–55. doi: 10.1007/s00125-020-05332-z (PMC7864814; doi:10.1007/s00125-020-05332-z)
Supplement: Supplementary file 1 — (PDF 643 kb) [file 125_2020_5332_MOESM1_ESM.pdf]

# Electronic supplementary material

## ESM Methods

**Edinburgh Hypoglycaemia Symptom Scale**
Edinburgh Hypoglycaemia Symptom Scale subdivisions and symptoms assessed. During the hypoglycaemic clamp, subjects were asked at 0, 95, 125, 170 and 215 min to rate the following symptoms on scales ranging from 1 (no symptoms) to 7 (maximal symptoms). Subjects were also invited to self-report at will, generating additional measurements.

| Autonomic | Neuroglycopenic | Malaise |
|---|---|---|
| Sweating | Confusion | Nausea |
| Palpitation | Drowsiness | Headache |
| Shaking | Odd behaviour | |
| Hunger | Speech difficulty | |
| | Incoordination | |

**Biochemical measurements**
A hexokinase method (Architect, Abbott) was used for glucose analysis. For hormones, the following analyses were used: insulin, cortisol and c-peptide (CobasE, Roche), ACTH and GH (Immulite 2000XPi, Siemens Healthcare Global). Glucagon was measured with ELISA (#10-1271-01, Mercodia, Uppsala, Sweden). Non-esterified fatty acids were measured with the Free Fatty Acid Fluorometric Assay Kit (Cayman Chemical, Ann Arbor, MI). Glycerol was measured with Free Glycerol reagent (Sigma-Aldrich, St. Louis, MO). All assays, absorbance or fluorescence, were read using a microplate reader (SpectraMax iD3, Molecular Devices, Berkshire, United Kingdom). Levels below 0.25 pmol/l of glucagon and 0.04 pmol/l of GH were expressed as 0.25 pmol/l and 0.04 pmol/l respectively.

**Heart rate variability analysis**
Heartbeats were automatically detected in the recorded ECG:s and the corresponding R-R intervals were determined, which was followed by manual inspection to remove errors due to falsely detected beats. Fluctuation in R-R intervals due to spurious arrhythmic beats were removed by interpolation. R–R intervals were transformed into evenly sampled (2 Hz) data by cubic spline interpolation. HRV was analysed by power spectrum analysis using Welch's periodogram method. The total spectral power ($P_{TOT}$) was calculated over consecutive 5-minute periods from the complete recording, as well as the power of the low-frequency ($P_{LF}$, 0.04-0.15 Hz) and high-frequency ($P_{HF}$, 0.15-0.50 Hz) spectral components. All spectral indices were log-transformed (base 10).

**Statistical analysis**
The weighted average definition of the median and percentiles was used throughout. For haemodynamic and biochemical measurements, the area under the curve (AUC) for the hypoglycaemic phase (80-185 min in HYPO) and the hyperglycaemic phase (30-165 min in HYPER) was calculated using the trapezoid method based on the actual time points of the measurements.  Change of area under the curve (△AUC) was calculated using those

measured levels after subtracting baseline levels (at 80 and 30 min of HYPO and HYPER, respectively). In the hyperglycaemic clamps, due to subject-variant glucose levels and some counterregulatory hormone levels decreasing toward a limit of detection, △AUCs of hormones were normalised and expressed as % in relation to baseline. AUC calculations were performed using Excel for Mac version 16.41 (Microsoft Corp, Redmond, WA, USA). Both the AUC and the △AUC were compared between the groups. For ESS scores, the peak scores from 80-185 min in HYPO and the difference between peak and baseline scores were compared. For HRV indices, the means for the euglycaemic/isoglycaemic phase, the hypoglycaemic/hyperglycaemic phase and the difference between these phases were calculated and compared separately.

For multilinear regression analyses, the △AUC of hormones and △means of HRV indices (euglycaemia to last stage of hypoglycaemia and isoglycaemia to hyperglycaemia) were chosen as dependent variables. Candidate predictors included the categorical variable sex (coded as dummy variable, female=0, male=1) and the continuous variables age, BMI, Waist-Hip Ratio (WHR), body fat percentage, fasting glucose, HbA1c, M-value and HOMA-IR. Guided by Spearman's Rank correlations with $p < 0.10$, BMI, fasting glucose and M-value were included as independent variables in the main multilinear regression analyses. Enter method was used. All models were exploratively adjusted for the $AUC_{Glucose}$, age and/or gender if deemed appropriate but this did not lead to reliable models that altered the results. For glucagon response, explorative adjustment for $AUC_{Insulin}$ was carried out, since there were slightly different clamp insulin levels between groups and insulin suppresses glucagon secretion. Collinearity was assessed by the VIF (>3 indicating collinearity issues). Normal distribution of the residuals was deemed acceptable for all models after visual inspection of p-p plots. Homoscedasticity was deemed acceptable after visual inspection of scatterplots of residuals vs predicted values. No correction for multiple testing was made.

Linear mixed models were generated with log-transformed (base 10) hormone levels at the end of clamp phase as dependent variables. The mean glucose for the preceding 20 minutes and Group (recoded as a dummy variable, LO= 0, HI=1) were selected as fixed effects. For glucagon, additional models were constructed, with BMI and M-value as continuous fixed effects besides glucose. Interactions with glucose were included in all models. All fixed effects were centred to the grand mean before analysis. Subject and glucose were selected as random effects, and the covariance structure was set to Variance Components.

## ESM Results

In interim analyses, we observed that the predetermined glucose infusion rate (200 mg/kg/min) during recovery from the hypoglycaemic clamp resulted in a higher GIR/FFM in HI vs LO which may have explained the faster glucose normalization in HI (a). Therefore, the infusion rate was adjusted for lean body mass (300 mg $kgFFM^{-1} h^{-1}$). Eight participants were subject to this change (5 LO, 3 HI). Glucose normalisation still occurred faster in HI vs LO upon visual inspection.

**ESM Table 1. Metabolic and hormonal responses during hypoglycaemic and hyperglycaemic clamps**

| Hypoglycaemic Clamps | | | | | Hyperglycaemic Clamps | | | |
|---|---|---|---|---|---|---|---|---|
| **Variable** | **Measurement** | **LO** | **HI** | **p** | **Measurement** | **LO** | **HI** | **p** |
| Glucose | $\text{Mean}_{\text{Eu}}$<br>(mmol/l) | 4.9<br>(4.8, 5.0) | 5.0<br>(4.9, 5.2) | 0.089 | $\text{Mean}_{\text{Iso}}$<br>(mmol/l) | 4.8<br>(4.4, 5.4) | 5.3<br>(4.9, 5.5) | **0.037** |
| | $\text{Mean}_{3.8}$<br>(mmol/l) | 3.8<br>(3.7, 3.9) | 3.9<br>(3.9, 4.2) | **0.002** | $\text{Mean}_{+3}$<br>(mmol/l) | 7.9<br>(7.2, 8.6) | 8.1<br>(7.9, 8.5) | 0.290 |
| | $\text{Mean}_{3.2}$<br>(mmol/l) | 3.0<br>(2.8, 3.1) | 3.1<br>(3.1, 3.2) | **0.008** | $\text{Mean}_{+6}$<br>(mmol/l) | 10.7<br>(10.3, 11.2) | 11.2<br>(10.9, 11.7) | 0.051 |
| | $\text{Mean}_{2.7}$<br>(mmol/l) | 2.8<br>(2.7, 2.8) | 2.9<br>(2.7, 3.1) | 0.187 | $\text{Mean}_{+9}$<br>(mmol/l) | 13.2<br>(12.9, 13.9) | 13.9<br>(13.7, 14.3) | **0.018** |
| | $\text{AUC}_{\text{Hypo}}$<br>(mmol/l x min) | 338.0<br>(333.5, 344.8) | 351.3<br>(345.8, 362.9) | **0.001** | $\text{AUC}_{\text{Hyper}}$<br>(mmol/l x min) | 1401.3<br>(1341.8, 1491.0) | 1481.9<br>(1448.0, 1528.5) | 0.063 |
| GIR/FFM | $\text{GIR}_{\text{Hypo}}$ (mg<br>$\text{kgFFM}^{-1}\ \text{min}^{-1}$) | 6.87<br>(5.75, 8.95) | 3.53<br>(1.85, 5.86) | **<0.001** | $\text{GIR}_{\text{Hyper}}$ (mg<br>$\text{kgFFM}^{-1}\ \text{min}^{-1}$) | 11.27<br>(9.41, 15.39) | 11.42<br>(9.30, 12.27) | 0.591 |
| M-value | $\text{GIR}_{\text{40-80min}}$ (mg<br>$\text{kgFFM}^{-1}\ \text{min}^{-1}$) | 11.49<br>(9.65, 15.47) | 6.29<br>(4.36, 10.32) | **0.007** | | | | |
| NEFA | $\text{AUC}_{\text{Hypo}}$<br>(µmol/l x min) | 1274<br>(920, 2750) | 4953<br>(1648, 32,876) | **0.004** | $\text{AUC}_{\text{Hyper}}$<br>(µmol/l x min) | 9418<br>(5784, 12,221) | 11274<br>(9558, 49,986) | **0.023** |
| | $\triangle\text{AUC}_{\text{Hypo}}$<br>(µmol/l x min) | 911<br>(225, 1554) | 503<br>(-385, 795) | 0.267 | $\triangle\text{AUC}_{\text{Hyper}}$<br>(µmol/l x min) | -18,066<br>(-22,970, -12,415) | -13670<br>(-18647, -10,866) | 0.310 |
| Glycerol | $\text{AUC}_{\text{Hypo}}$<br>(µmol/l x min) | 4002<br>(3423, 5308) | 4759<br>(4094, 6798) | 0.067 | $\text{AUC}_{\text{Hyper}}$<br>(µmol/l x min) | 4697<br>(4427, 5273) | 8342<br>(6838, 9355) | **<0.001** |
| | $\triangle\text{AUC}_{\text{Hypo}}$<br>(µmol/l x min) | 208<br>(-363, 844) | -380<br>(-1914, 419) | 0.217 | $\triangle\text{AUC}_{\text{Hyper}}$<br>(µmol/l x min) | -4412<br>(-6037, -2907) | -4016<br>(-6269, -3592) | 1.000 |
| Insulin | $\text{AUC}_{\text{Hypo}}$<br>(pmol/l x min) | 74,121<br>(60,630, 82,194) | 86,726<br>(72,471, 105,644) | **0.015** | $\text{AUC}_{\text{Hyper}}$<br>(pmol/l x min) | 27,789<br>(26,082, 47,191) | 80,642<br>(50,268, 111,775) | **0.008** |
| C-peptide | $\text{AUC}_{\text{Hypo}}$<br>(nmol/l x min) | 20.63<br>(18.45, 30.75) | 38.25<br>(24.75, 57.75) | **0.002** | $\text{AUC}_{\text{Hyper}}$<br>(nmol/l x min) | 272.25<br>(241.25, 308.00) | 398.13<br>(307.44, 498.19) | **0.004** |

| | | | | | | | | |
|---|---|---|---|---|---|---|---|---|
| Glucagon | $AUC_{Hypo}$ (pmol/l x min) | 1431 (1130, 1730) | 1559 (873, 2072) | 0.967 | $AUC_{Hyper}$ (pmol/l x min) | 390 (244, 476) | 547 (337, 618) | 0.085 |
| | $\triangle AUC_{Hypo}$ (pmol/l x min) | 1006 (666, 1517) | 1007 (530, 1422) | 0.713 | $\triangle AUC_{Hyper}$ (%) | -73.0 (-80.8, -68.2) | -63.4 (-71.4, -60.1) | **0.010** |
| Cortisol | $AUC_{Hypo}$ (nmol/l x min) | 28,545 (21,975, 38805) | 32,430 (21,308, 40,898) | 0.512 | $AUC_{Hyper}$ (nmol/l x min) | 28,133 (20,585, 39,408) | 25,038 (20,584, 30,087) | 0.425 |
| | $\triangle AUC_{Hypo}$ (nmol/l x min) | 4793 (-1200, 11498) | 12383 (7058, 15705) | 0.050 | $\triangle AUC_{Hyper}$ (%) | -4.2 (-18.2, 8.5) | 0.6 (-8.6, 66.0) | 0.310 |
| ACTH | $AUC_{Hypo}$ (pmol/l x min) | 417.0 (325.5, 859.5) | 741.0 (657.0, 1088.8) | **0.037** | $AUC_{Hyper}$ (pmol/l x min) | 362.3 (267.8, 479.3) | 416.3 (289.7, 588.9) | 0.331 |
| | $\triangle AUC_{Hypo}$ (pmol/l x min) | 162.0 (125.3, 397.5) | 437.3 (362.9, 634.5) | **0.021** | $\triangle AUC_{Hyper}$ (%) | 11.8 (4.4, 24.7) | 21.5 (9.1, 53.5) | 0.290 |
| GH | $AUC_{Hypo}$ (µg/l x min) | 491.2 (143.0, 866.3) | 309.7 (110.6, 679.5) | 0.250 | $AUC_{Hyper}$ (µg/l x min ) | 84.6 (16.2, 310.1) | 28.7 (9.7, 50.9) | **0.037** |
| | $\triangle AUC_{Hypo}$ min (µg/l x min ) | 226.7 (77.8, 632.7) | 238.2 (84.3, 553.5) | 0.967 | $\triangle AUC_{Hyper}$ (%) | -43.2 (-56.2, -6.7) | -54.5 (-71.7, 0.0) | 0.505 |

Data presented as median (25$^{th}$ percentile, 75$^{th}$ percentile). P-values refer to comparisons between groups HI and LO. Significant p-values are shown in **bold**. No corrections for multiple testing.
AUC= Area Under the Curve, △AUC= delta AUC, GIR= Glucose Infusion Rate, FFM= Fat-Free Mass (by bioimpedance), FFA= free fatty acids, GH= growth hormone.
Subscriptions: $_{Eu}$=Euglycaemic phase, 0-80 min of HYPO, $_{3.8}$=80-110 min of HYPO, $_{3.2}$= 110-155 min of HYPO, $_{2.7}$=155-185 min of HYPO. $_{Iso}$=Isoglycaemic phase, 0-30 min of HYPER, $_{+3}$=30-75 min of HYPER, $_{+6}$=75-120 min of HYPER, $_{+9}$=120-165 min of HYPER. $_{Hypo}$=80-185 min of HYPO, $_{Hyper}$= 30-165 min of HYPER.

**ESM Table 2. Haemodynamic and heart rate variability responses during hypoglycaemic and hyperglycaemic clamps**

| | Hypoglycaemic Clamps | | | | Hyperglycaemic Clamps | | | |
|---|---|---|---|---|---|---|---|---|
| **Variable** | **Measure** | **LO** | **HI** | **p** | **Measure** | **LO** | **HI** | **p** |
| SBP | $AUC_{Hypo}$ (mmHg x min) | 12,090 (11,205, 12,480) | 12,690 (12,210, 13,335) | **0.036** | $AUC_{Hyper}$ (mmHg x min) | 15,310 (14,450, 16,460) | 16,275 (15,104, 17,287) | 0.089 |
| | $\triangle AUC_{Hypo}$ (mmHg x min) | -345 (-720, 330) | -262.5 (-525, -60) | 0.351 | $\triangle AUC_{Hyper}$ (mmHg x min) | -240 (-620, 185) | -91 (-636, 432) | 0.879 |
| DBP | $AUC_{Hypo}$ (mmHg x min) | 7065 (6495, 7740) | 8010 (7290, 8415) | **0.015** | $AUC_{Hyper}$ (mmHg x min) | 9630 (9385, 10,468) | 10,474 (9752, 10,996) | 0.111 |
| | $\triangle AUC_{Hypo}$ (mmHg x min) | -450 (-855, -227) | -345 (-810, -195) | 0.648 | $\triangle AUC_{Hyper}$ (mmHg x min) | -135 (-740, -65) | -429 (-714, -176) | 0.371 |
| HR* | $AUC_{Hypo}$ (bpm x min) | 7140 (6405, 7800) | 7005 (6330, 8490) | 0.678 | $AUC_{Hyper}$ (bpm x min) | 7835 (7060, 9090) | 8403 (7575, 1293) | 0.315 |
| | $\triangle AUC_{Hypo}$ (bpm x min) | 375 (60, 675) | 90 (0, 495) | 0.146 | $\triangle AUC_{Hyper}$ (bpm x min) | -90 (-453, 328) | 45 (-411, 505) | 0.694 |
| RR* | $Mean_{EU}$ (s) | 0.85 (0.82, 0.95) | 0.91 (0.78, 1.00) | 0.959 | $Mean_{Iso}$ (s) | 0.99 (0.87, 1.09) | 0.94 (0.81, 1.02) | 0.402 |
| | $Mean_{Hypo}$ (s) | 0.82 (0.74, 0.89) | 0.89 (0.74, 0.96) | 0.507 | $Mean_{Hyper}$ (s) | 0.98 (0.86, 1.12) | 0.94 (0.81, 1.06) | 0.550 |
| | $\triangle Mean_{Hypo-Eu}$ (s) | -0.06 (-0.11, 0.00) | -0.02 (-0.04, 0.02) | 0.097 | $\triangle Mean_{Hyper-Iso}$ (s) | -0.02 (-0.04, 0.04) | -0.00 (-0.02, 0.03) | 0.650 |
| | $Mean_{2.7}$ (s) | 0.77 (0.71, 0.86) | 0.85 (0.75, 0.92) | 0.237 | | | | |
| | $\triangle Mean_{2.7-Eu}$ (s) | -0.12 (-0.14, -0.03) | -0.05 (-0.07, 0.03) | **0.047** | | | | |
| $P_{TOT}$ | $Mean_{Eu}$ ($ms^2$, log) | 3.37 (3.32, 3.69) | 3.46 (3.34, 3.57) | 0.838 | $Mean_{Iso}$ ($ms^2$, log) | 3.69 (3.50, 3.75) | 3.51 (3.34, 3.73) | 0.302 |
| | $Mean_{Hypo}$ | 3.39 | 3.38 | 0.799 | $Mean_{Hyper}$ | 3.71 | 3.53 | 0.239 |

| | | | | | | | | |
|---|---|---|---|---|---|---|---|---|
| | (ms$^2$, log) | (3.18, 3.69) | (3.27, 3.60) | | (ms$^2$, log) | (3.45, 3.94) | (3.40, 3.73) | |
| | △Mean$_{Hypo\text{-}Eu}$<br>(ms$^2$ ,log) | -0.05<br>(-0.17, 0.03) | -0.01<br>(-0.08, 0.08) | 0.281 | △Mean$_{Hyper\text{-}Iso}$<br>(ms$^2$ ,log) | 0.01<br>(-0.06, 0.07) | -0.01<br>(-0.03, 0.03) | 0.793 |
| | Mean$_{2.7}$<br>(ms$^2$, log) | 3.20<br>(3.04, 3.43) | 3.41<br>(3.25, 3.80) | 0.061 | | | | |
| | △Mean$_{2.7\text{-}Eu}$<br>(ms$^2$, log) | -0.23<br>(-0.39, -0.03) | -0.04<br>(-0.16, 0.31) | **0.047** | | | | |
| $P_{LF}$ | Mean$_{Eu}$<br>(ms$^2$, log) | 2.99<br>(2.76, 3.28) | 2.89<br>(2.81, 3.07) | 0.384 | Mean$_{Iso}$<br>(ms$^2$, log) | 3.26<br>(3.01, 3.34) | 2.97<br>(2.85, 3.16) | 0.155 |
| | Mean$_{Hypo}$<br>(ms$^2$, log) | 2.87<br>(2.71, 3.13) | 2.86<br>(2.62, 3.14) | 0.838 | Mean$_{Hyper}$<br>(ms$^2$, log) | 3.24<br>(3.01, 3.44) | 2.98<br>(2.85, 3.21) | 0.076 |
| | △Mean$_{Hypo\text{-}Eu}$<br>(ms$^2$ ,log) | -0.09<br>(-0.16, -0.06) | -0.04<br>(-0.10, 0.04) | 0.097 | △Mean$_{Hyper\text{-}Iso}$<br>(ms$^2$ ,log) | 0.02<br>(-0.03, 0.07) | -0.01<br>(-0.09, 0.08) | 0.550 |
| | Mean$_{2.7}$<br>(ms$^2$, log) | 2.66<br>(2.55, 2.84) | 2.85<br>(2.67, 3.26) | 0.121 | | | | |
| | △Mean$_{2.7\text{-}Eu}$<br>(ms$^2$, log) | -0.31<br>(-0.40, -0.11) | -0.11<br>(-0.23, 0.22) | **0.018** | | | | |
| $P_{HF}$ | Mean$_{Eu}$<br>(ms$^2$, log) | 2.59<br>(2.26, 2.83) | 2.55<br>(2.15, 2.80) | 0.683 | Mean$_{Iso}$<br>(ms$^2$, log) | 2.78<br>(2.50, 3.09) | 2.59<br>(2.47, 3.12) | 0.685 |
| | Mean$_{Hypo}$<br>(ms$^2$, log) | 2.46<br>(2.12, 2.84) | 2.52<br>(2.10, 2.81) | 0.838 | Mean$_{Hyper}$<br>(ms$^2$, log) | 2.93<br>(2.39, 3.10) | 2.81<br>(2.51, 3.07) | 0.981 |
| | △Mean$_{Hypo\text{-}Eu}$<br>(ms$^2$ ,log) | -0.12<br>(-0.21, -0.05) | -0.01<br>(-0.13, 0.12) | 0.097 | △Mean$_{Hyper\text{-}Iso}$<br>(ms$^2$ ,log) | -0.03<br>(-0.09, 0.06) | 0.06<br>(-0.04, 0.15) | 0.220 |
| | Mean$_{2.7}$<br>(ms$^2$, log) | 2.12<br>(1.97, 2.60) | 2.53<br>(2.01, 2.88) | 0.164 | | | | |
| | △Mean$_{2.7\text{-}Eu}$<br>(ms$^2$, log) | -0.22<br>(-0.48, -0.13) | 0.04<br>(-0.35, 0.19) | **0.024** | | | | |
| $P_{LF}/P_{HF}$ | Mean$_{Eu}$<br>(ratio, log) | 0.45<br>(0.39, 0.50) | 0.35<br>(0.12, 0.71) | 0.574 | Mean$_{Iso}$<br>(ratio, log) | 0.33<br>(0.28, 0.51) | 0.25<br>(0.15, 0.51) | 0.202 |
| | Mean$_{Hypo}$<br>(ratio, log) | 0.45<br>(0.39, 0.61) | 0.30<br>(0.21, 0.70) | 0.384 | Mean$_{Hyper}$<br>(ratio, log) | 0.38<br>(0.26, 0.57) | 0.15<br>(0.08, 0.30) | **0.005** |

| | | | | | | | |
|---|---|---|---|---|---|---|---|
| $\triangle Mean_{Hypo-Eu}$ (ratio ,log) | 0.03 (-0.04, 0.08) | -0.04 (-0.12, 0.02) | 0.180 | $\triangle Mean_{Hyper-Iso}$ (ratio, log) | 0.04 (-0.01, 0.06) | -0.06 (-0.13, 0.00) | **0.011** |
| $Mean_{2.7}$ (ratio, log) | 0.53 (0.38, 0.65) | 0.37 (0.18, 0.66) | 0.413 | | | | |
| $\triangle Mean_{2.7-Eu}$ (ratio, log) | 0.03 (-0.02, 0.15) | -0.02 (-0.11, 0.08) | 0.413 | | | | |

Data presented as median (25$^{th}$ percentile,75$^{th}$ percentile). P-values refer to comparisons between groups HI and LO. Significant p-values are shown in **bold**. No corrections for multiple testing.
HR=Heart Rate, SBP=Systolic Blood Pressure, DBP=Diastolic Blood Pressure, RR= R-R interval, $P_{Tot}$= Total Power, $P_{HF}$=Power of high-frequency component, $P_{LF}$=Power of low-frequency component. Subscriptions: $_{Eu}$=Euglycaemic phase, 0-80 min of HYPO, , $_{2.7}$=155-185 min of HYPO. $_{Iso}$=Isoglycaemic phase, 0-30 min of HYPER, $_{Hypo}$=80-185 min of HYPO, $_{Hyper}$= 30-165 min of HYPER. $_{Hypo-Eu}$=Difference between $_{Hypo}$ and $_{Eu}$, $_{2.7-Eu}$=Difference between $_{2.7}$ and $_{Eu}$, $_{Hyper-Iso}$= Difference between $_{Hyper}$ and $_{Iso}$.
*While RR is the inverse of HR, they were assessed differently and on different timepoints, explaining minor discrepancies in the results.

**ESM Table 3. Correlations and multilinear regressions between Edinburgh Hypoglycaemia Symptom Scale scores, baseline subject characteristics and neuroendocrine responses.**

| | **Spearman's Rank Correlations** | **Multilinear regression**⋆ |
|---|---|---|
| | **ΔESS** | **ΔESS** |
| $R^2$, p | NA | **0.253, 0.023** |
| **Variable** | $r_s$, p | $\beta$, p |
| Age | -0.169, 0.372 | NA |
| Gender | -0.171, 0.368 | NA |
| BMI | 0.324, 0.081 | 0.168, 0.354 |
| WHR | 0.131, 0.490 | NA |
| Body fat (%) | 0.276, 0.139 | NA |
| Fasting Glucose | -0.288, 0.123 | NA |
| HbA1c | -0.173, 0.361 | NA |
| M-value | -0.141, 0.459 | NA |
| HOMA-IR | 0.318, 0.087 | NA† |
| $AUC_{Glucose}$ | 0.225, 0.232 | NA |
| $\triangle AUC_{Glucagon}$ | 0.191, 0.312 | NA |
| $\triangle AUC_{Cortisol}$ | **0.411, 0.024** | **0.426, 0.024** |
| $\triangle AUC_{ACTH}$ | **0.375, 0.041** | NA |
| $\triangle AUC_{GH}$ | 0.266, 0.156 | NA |
| $\Delta P_{HF}Mean_{2.7\text{-}Eu}$ | -0.001, 0.995 | NA |
| $\Delta P_{LFHF}Mean_{2.7\text{-}Eu}$ | 0.140, 0.496 | NA |

Significant correlations are shown in **bold**. Pooled data (n=30, ⋆one outlier excluded). ESS= Edinburgh Hypoglycaemia Symptom Scale, p= p-value, NA= Not Applicable/Not Analysed, $r_s$= Spearman's rho coefficient, $\beta$ = standardised coefficient, WHR= Waist-Hip ratio, AUC= area under the curve, △AUC delta area under the curve, $\Delta P_{HF}Mean_{2.7\text{-}Eu}$ = difference in the mean of the power of the high-frequency component between the hypoglycaemic stage with target glucose 2.7 mmol/l (155-185 min) and the euglycaemic phase (0-80 min), $\Delta P_{LFHF}Mean_{2.7\text{-}Eu}$ = difference in the mean of the ratio of the power of the low-frequency component and the power of high-frequency component between the hypoglycaemic stage with target glucose 2.7 mmol/l (155-185 min) and the euglycaemic phase (0-80 min).

**ESM Figure 1. Scatterplot of ΔESS vs BMI and cortisol response to hypoglycaemia**

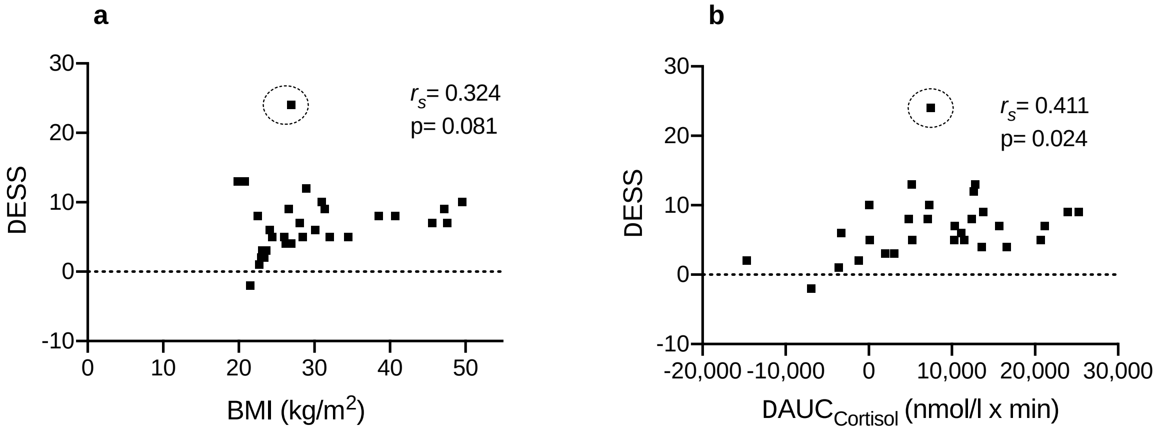


ΔESS= Change of Edinburgh Hypoglycaemia Symptom Scale scores from baseline to peak, $r_s$= Spearman’s Rho Coefficient, p= p-value of Spearman’s Rank Correlation analysis, $\triangle AUC_{Cortisol}$= delta area under the curve of cortisol during hypoglycaemic phase (80-185 min). Dashed circle indicates outlier removed from multilinear regression in ESM Table 3.
.
